# Morning Glory Disc Anomaly With Ipsilateral Carotid Vasculopathy: MRI Characteristics and Clinical Implications

**DOI:** 10.7759/cureus.93932

**Published:** 2025-10-06

**Authors:** Nivedita Radder, Shrinivas Radder

**Affiliations:** 1 Diagnostic Radiology, University of Arkansas for Medical Sciences, Little Rock, USA; 2 Diagnostic Radiology/Pediatric Radiology, University of Arkansas for Medical Sciences/Arkansas Children's Hospital, Little Rock, USA

**Keywords:** cerebrovascular anomalies, morning glory disc anomaly, moyamoya disease, mri, optic nerve malformation, pediatric ophthalmology

## Abstract

Morning glory disc anomaly (MGDA) is a rare developmental malformation of the optic nerve that can complicate the diagnosis and treatment of children. We describe a case of a three-year-old girl who presented with a reduction of vision and an exotropia of the right eye. Fundoscopic observation revealed evidence of MGDA, which was confirmed through magnetic resonance imaging (MRI). MRI showed the characteristic triad of orbital appearances: a funnel-shaped morphologic structure of the posterior optic disc and elevation of the adjacent retinal surface, abnormal tissue surrounding the ipsilateral optic nerve distal part with obliteration of the surrounding subarachnoid spaces, and discontinuity of the uveoscleral coat. Post-contrast imaging revealed enhancement in the distal retrobulbar optic nerve area, likely representing displaced choroidal tissue accompanied by glial and fibrous proliferation. Notably, MR angiography showed narrowing of the right internal carotid artery segments (petrous, cavernous, and supraclinoid) with a hypoplastic right A1 segment. This represents an intermediate vascular phenotype--significant vasculopathy without the collateral vessels that define Moyamoya disease. This case highlights the importance of comprehensive neuroimaging in MGDA patients, as early identification of associated vascular anomalies is crucial for appropriate surveillance and management. The consistent MRI findings described can aid in differentiating MGDA from other ocular anomalies, particularly optic nerve coloboma, which has different genetic and prognostic implications.

## Introduction

Morning glory disc anomaly (MGDA) is an unusual developmental anomaly of the optic nerve first described by Kindler in 1970, who observed its resemblance to the morning glory flower [1,2]. This rare disorder, occurring in approximately 1 in 10,000 births, is characterized by an enlarged and depressed optic disc having a funnel-like structure of the posterior globe, with three main funduscopic features: an enlarged excavation, pigmentary alterations surrounding the excavation of the chorioretina, and a centrally located glial tuft [3,4].

The clinical significance of MGDA extends beyond its ocular manifestations. These patients face substantial visual impairment, with visual acuity typically ranging from 20/200 to light perception, and are at increased risk of complications, particularly retinal detachment, which occurs in up to 38% of cases [5,6]. Furthermore, MGDA is frequently associated with intracranial anomalies, particularly cerebrovascular anomalies, including Moyamoya disease (20-45% of cases), midline craniofacial defects, and basal encephaloceles [7-9].

Although the diagnosis is typically made clinically through funduscopic examination, neuroimaging plays an increasingly vital role in both confirming the diagnosis and screening for associated abnormalities. This is particularly important when physical examination is limited in young children or when differentiating MGDA from other conditions, such as optic nerve coloboma, which carries different genetic counseling implications [10,11].

## Case presentation

The parents of a three-year-old girl presented to our pediatric ophthalmology clinic, having noticed their daughter's right eye turning outward over the past several months. The mother reported that the child appeared to bump into objects on her right side and occasionally tilted her head when reading books. There was no history of headache, eye redness, or gait imbalance. The patient was born at full term via normal vaginal delivery with no perinatal complications. Developmental milestones were age-appropriate (Denver Developmental Screening Test), and there was no family history of ocular or neurological disorders.

On examination, the child was alert and interactive. Neurologic assessment revealed intact cranial nerves (except for the visual deficit), normal tone and reflexes, and no focal neurological deficits. Cardiovascular evaluation by pediatric cardiology showed normal heart sounds, no murmurs, normal peripheral pulses, and blood pressure for age. ECG and echocardiogram were normal with no signs of cerebral hypoperfusion. Systemic examination revealed normal growth parameters (50th percentile), no dysmorphic features or midline facial defects, normal hearing screen, and normal renal ultrasound (performed to exclude syndromic associations).

Visual acuity testing revealed markedly decreased vision in the right eye, with the child only able to perceive hand movements. The left eye demonstrated age-appropriate visual acuity. Ocular motility examination revealed a 15-degree exotropia of the right eye. Pupillary examination showed a relative afferent pupillary defect on the right. Anterior segment examination was unremarkable bilaterally, showing clear corneas, normal anterior chamber depth, clear lenses without cataract, and normal iris architecture without colobomas.

**Figure 1:**
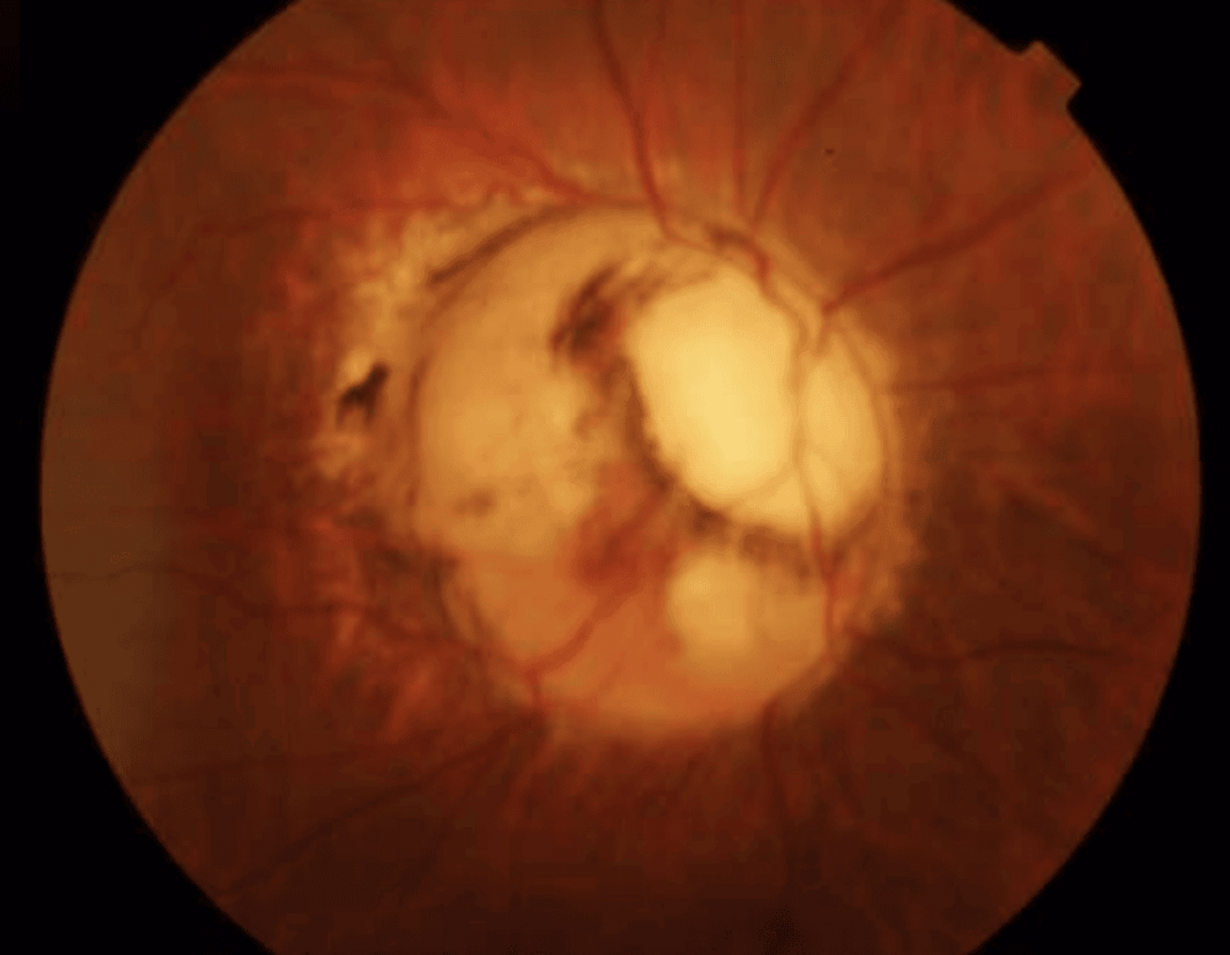
Dilated fundoscopy of the right eye reveals an enlarged and excavated optic disc with a funnel-shaped structure, filled with white hyperplastic glial tissue. The vessels of the retina appeared in a strange radial formation at the extremes of the abnormal disc.

Dilated fundoscopic examination of the right eye revealed striking abnormalities consistent with MGDA (Figure 1). The optic disc appeared enlarged and excavated with a funnel-shaped configuration. A characteristic annulus of chorioretinal pigmentary disturbance surrounded the disc, and a central white glial tuft was visible overlying the disc. The retinal vessels emerged in an unusual radial pattern from the periphery of the anomalous disc. The left fundus examination was entirely normal.

Genetic counseling was offered to the family. We discussed that MGDA is almost universally sporadic with negligible recurrence risk, while optic nerve coloboma can be familial and associated with genetic syndromes. The parents declined genetic testing given the pathognomonic MRI findings confirming MGDA, the absence of syndromic features, and understanding of the sporadic nature with minimal recurrence risk.

Given the fundoscopic findings and the need to evaluate for associated intracranial abnormalities, a comprehensive MRI of the brain and orbits with MR angiography was performed.

Imaging findings

**Figure 2:**
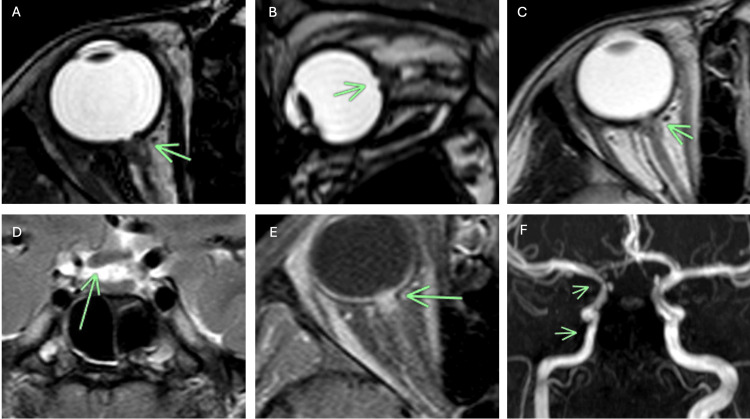
Imaging findings in a patient with right orbit morning glory disc anomaly (MGDA) Balanced fast field echo (BFFE) MR sequence in the axial plane (A) and sagittal plane (B) through the right orbit shows a funnel-shaped morphologic structure of the optic disc (arrow) with uplift of the adjacent retinal surface. This image also shows abnormal tissue, which is associated with the terminal of the intraorbital segment of the right optic nerve and subarachnoid space compression of this level (arrow). The vascular layer of uveosclerosis at the site of optic nerve entry is also intermittent. C) The axial T2-weighted image revealed focal effacement of the perioptic CSF space caused by abnormal thickening of the soft tissue at the end of the intraorbital segment of the right optic nerve(arrow). (D) The T2-weighted coronal image represents asymmetric thickening of the right optic chiasm (arrow). (E) The axial T1-post contrast image shows abnormal enhancement in the distal retrobulbar area of the right optic nerve, probably due to displaced choroidal tissue, which is accompanied by glial, fibrous, and pigment epithelial proliferation. (F) MRI angiography across the Circle of Willis showed severe vascular abnormalities of the right internal carotid artery system (ICA). The intracranial ICA right side segments, including the petrous, cavernous, and supraclinoid, appeared to be narrow. The right anterior cerebral artery (A1) was hypoplastic. The rest of the circle of Willis and the vertebrobasilar system looked normal. No evidence of abnormal vascular collateralization, indicating the presence of Moyamoya disease, was found at this point.

MRI of the orbits demonstrated pathognomonic features of MGDA in the right eye. High-resolution T2-weighted images revealed a funnel-shaped morphologic pattern of the posterior optic disc with marked elevation of the adjacent retinal surface. The normal hypointense ring representing the uveoscleral coat (sclera, choroid, and lamina cribrosa) showed clear discontinuity at the optic nerve insertion, in stark contrast to the intact appearance on the left (Figures 2A-2B).

Abnormal soft tissue was identified along the distal intraorbital segment of the right optic nerve, causing focal effacement of the perioptic subarachnoid space. This tissue demonstrated intermediate signal intensity on T1-weighted images and mild hyperintensity on T2-weighted sequences (Figure 2C). The right optic chiasm appeared asymmetrically small (Figure 2D). Post-contrast T1-weighted images revealed enhancement in the distal retrobulbar region of the right optic nerve, likely representing displaced choroidal tissue with associated glial, fibrous, and pigment epithelial proliferation (Figure 2E).

Diffusion-weighted imaging (DWI) showed no evidence of acute or chronic ischemic changes in the brain parenchyma. Perfusion imaging was not performed at initial presentation, as the patient had no clinical symptoms of cerebral hypoperfusion and the degree of narrowing without collaterals suggested compensated flow.

MR angiography revealed significant vascular abnormalities involving the right internal carotid artery (ICA) system (Figure 2F). There was narrowing of the petrous, cavernous, and supraclinoid segments of the right ICA. The right A1 segment of the anterior cerebral artery appeared hypoplastic. Importantly, no abnormal collateral vessels or "puff of smoke" appearance characteristic of Moyamoya disease were identified. The remainder of the circle of Willis and the vertebrobasilar system appeared normal. The brain parenchyma showed no signal abnormalities, and there was no evidence of midline defects or encephaloceles.

## Discussion

Our case demonstrates the characteristic MRI findings of MGDA and highlights the importance of comprehensive neuroimaging in these patients. The imaging features we observed align with previous reports in the literature, confirming three consistent orbital findings: the funnel-shaped optic disc morphology, abnormal perioptic soft tissue with CSF effacement, and uveoscleral coat discontinuity [3,10,12].

The pathogenesis of MGDA remains incompletely understood, though current evidence suggests a primary defect in posterior scleral development during embryogenesis. Histopathologic studies have demonstrated that the normal mature sclera is replaced by disorganized spindle cells resembling embryonic mesenchyme, resulting in failure of normal fusion and allowing herniation of the optic disc, lamina cribrosa, peripapillary retina, and choroid posteriorly [13,14]. This theory explains our MRI observations: the uveoscleral discontinuity represents the primary defect, while the abnormal perioptic tissue likely reflects evaginated choroid and peripapillary retina with associated CSF effacement.

The enhancement pattern observed in our patient deserves particular attention. The post-contrast enhancement in the distal optic nerve region likely represents displaced choroidal tissue, which is inherently vascular, possibly combined with reactive gliosis and fibrovascular proliferation [3,15]. This finding, while not universally present, can help confirm the diagnosis when observed.

Vascular findings and their significance

Our case demonstrates an important intermediate vascular phenotype - significant ICA narrowing with A1 hypoplasia but without the collateral vessels that define Moyamoya disease. This differs from classic Moyamoya, which features progressive stenosis at the ICA terminus with extensive basal collaterals creating the characteristic "puff of smoke" appearance. Our patient's findings may represent an early stage in vascular progression, as documented by Hanson et al. and Massaro et al., who reported patients with initial carotid abnormalities that later evolved into full Moyamoya disease [16,17]. This highlights the importance of early vascular screening even in asymptomatic MGDA patients.

Understanding the embryologic relationship

The association between MGDA and carotid vasculopathy can be understood through their shared developmental timeline. During the fourth week of embryonic development, two critical events occur simultaneously in close proximity: the optic vesicle buds out from the developing brain to form future eye structures, and the internal carotid arteries begin forming immediately adjacent to these developing optic structures. Both processes rely on proper migration and differentiation of neural crest cells and mesenchymal tissue. This explains why a developmental insult during this critical period could damage both the optic nerve (causing MGDA) and the adjacent blood vessels (causing carotid abnormalities), resulting in the combination seen in up to 45% of MGDA patients [18].

The differential diagnosis for MGDA primarily includes optic nerve coloboma, which can present with similar clinical features. However, distinguishing between these entities is crucial: MGDA is almost universally sporadic, while optic nerve coloboma frequently shows familial clustering and may occur as part of multisystem syndromes such as CHARGE syndrome [11,19]. Our MRI findings, particularly the funnel-shaped disc morphology, perioptic soft tissue, and enhancement pattern, are highly specific for MGDA and have not been reported in coloboma [20].

Management and surveillance

Management of MGDA requires a multidisciplinary approach. All MGDA patients should have at least one neurology consultation for baseline assessment and counseling about warning symptoms. From an ophthalmologic perspective, patients need monitoring every three to six months for retinal detachment screening, which may occur due to abnormal communication between the subretinal and subarachnoid spaces [5,6]. Amblyopia therapy for the fellow eye is crucial to maximize visual potential.

Long-term follow-up plan

Our patient follows a comprehensive surveillance protocol designed to detect both ocular and vascular complications early. Ophthalmologic examinations are performed every three months to screen for retinal detachment, given the 38% lifetime risk in MGDA patients, with immediate evaluation if any visual symptoms develop. For vascular monitoring, MR angiography is scheduled every six months for the first two years, then annually if stable, with perfusion sequences incorporated into future studies based on reviewer recommendations. Neurology follow-up occurs every six months to assess for subclinical neurological changes. The threshold for neurosurgical referral includes the development of Moyamoya collaterals on imaging, clinical symptoms such as transient ischemic attacks or seizures, evidence of hypoperfusion on perfusion imaging, or progressive stenosis on serial studies. At the six-month follow-up visit, our patient remains neurologically asymptomatic with no progression of vasculopathy on repeat MRA and stable visual function, though continued vigilance is maintained given the potential for delayed vascular progression documented in the literature.

## Conclusions

This case highlights three essential points for clinicians managing MGDA. First, MRI demonstrates a pathognomonic triad of funnel-shaped optic disc, perioptic tissue abnormalities, and uveoscleral discontinuity that confirms the diagnosis and differentiates MGDA from optic nerve coloboma, thereby avoiding unnecessary genetic testing. Second, all MGDA patients require baseline MR angiography regardless of symptoms, as up to 45% harbor vascular anomalies that may be progressive yet initially asymptomatic, as demonstrated in our patient. Finally, establishing appropriate surveillance protocols is crucial--regular ophthalmologic monitoring for retinal detachment (38% lifetime risk) and serial neuroimaging for those with vascular findings can prevent devastating visual and neurological complications through early detection and timely intervention.
